# An Experimental Strategy for Capturing the Margins of Prepared Single Teeth with an Intraoral Scanner: A Prospective Clinical Study on 30 Patients

**DOI:** 10.3390/ijerph17020392

**Published:** 2020-01-07

**Authors:** Francesco Guido Mangano, Bidzina Margiani, Ivan Solop, Nadezhda Latuta, Oleg Admakin

**Affiliations:** 1 Private Practice, Gravedona, 22015 Como, Italy; 2 Department of Prevention and Communal Dentistry, Sechenov First Moscow State Medical University, 119991 Moscow, Russia; margiani.b@gmail.com (B.M.); solopivan@yandex.ru (I.S.); latuta.n@mail.ru (N.L.); admakin1966@mail.ru (O.A.)

**Keywords:** intraoral scanners, prepared teeth, margin line, monolithic zirconia crowns, clinical precision

## Abstract

*Purpose*: To present an experimental strategy for successfully capturing the margins of prepared single teeth with an intraoral scanner (IOS). *Methods*: The protocol was as follows: (1) an intraoral impression was captured with an IOS, without taking care of the visibility of the margins; (2) a partial analog impression was taken by means of a 3D-printed custom tray filled with polyvinylsiloxane light, after the removal of a retraction cord; (3) the hollow portion of the analog impression, with the preparation margins clearly visible, was scanned extraorally with the same IOS; (4) the scan of the analog impression was imported into computer-assisted-design (CAD) software, where its normals were inverted; (5) the scan with inverted normals was registered on the first intraoral scan, and replaced it; (6) the technician designed the final restoration, which was fabricated and delivered for application. The study outcomes were: (1) the marginal adaptation of the final crown; (2) the quality of interproximal contacts; and (3) the quality of occlusal contacts. *Results*: Thirty patients (18 males, 12 females; mean age 51.3 ± 11.6 years) were selected for this study. All these patients were restored with a monolithic translucent zirconia crown, fabricated following the aforementioned protocol. The clinical precision and the marginal adaptation of the crowns were optimal, interproximal contact points were perfect, and the only necessary adaptations were occlusal, with some minor precontacts that had to be polished. Conclusions: The present protocol seems to be compatible with the fabrication of clinically precise zirconia crowns. Further studies are needed to confirm these results.

## 1. Introduction

Today, the intraoral scanner (IOS) represents one of the most important innovations in dental technology introduced by the digital revolution [1,2]. In fact, the IOS allows to achieve accurate impressions of dental arches using the power of structured light; these optical impressions are subsequently used to design and manufacture, through milling, a whole series of prosthetic restorations, using computer-assisted-design/computer-assisted-manufacturing (CAD/CAM) techniques [1,2]. Optical impressions eliminate the need for conventional impressions with big trays and materials, which have always been unwelcome to patients [3]. In fact, the conventional impressions represent discomfort and stress for patients, especially if they have a strong gag reflex [3,4]. Further advantages of the use of the optical impressions are time-efficiency, the simplification of clinical procedures, and the possibility of eliminating plaster models. Now, with digital impressions, even more efficient communication with the technician and the patient is possible [2,5]. The two main limitations of IOS, to date, are the difficulty of recording sufficiently accurate impressions for designing and fabricating full-arch restorations [5,6,7,8,9], and the difficulty of correctly detecting the margins of prosthetic preparations in natural teeth, especially in cases of iuxtagingival or subgingival margins [10,11]. In the case of full-arch restorations, this limitation applies both to the impressions on natural teeth and those on implants, and it is determined by the difficulty in capturing with IOS and reconstructing in 3D an extended area such as that of a complete dental arch [6,7,8,9]. However, this issue is limited to this specific clinical application, and it does not prevent the clinician from taking sufficiently accurate impressions to design and fabricate single crowns and partial fixed prostheses (up to 4 or 5 elements) [12,13,14,15,16]. For the margins of prosthetic preparations of natural teeth, instead, the problem can be relevant also for single crowns, and represents today the greatest difficulty for clinicians who have IOS, all over the world [10,11]. In fact, when using IOS and optical impressions in natural teeth, it can be difficult to capture all the details of the margins of the prosthetic preparations, particularly if they are iuxta- or subgingival [2,10,11]. In fixed prostheses, it is not always possible to work with supragingival preparations. In the case, for example, of remakes of previous prosthetic crowns or of discromic abutments in an anterior area, it is inevitable that the preparation margin be moved juxta- or subgingivally [10,11]. With IOS, this can become a problem. In fact, the structured light does not possess the rheological properties of conventional impression materials, which are able to penetrate into the sulcus (suitably displaced by the placement of one or more retractor cords), physically detach the gingiva, and thus record all details of the preparation in the best possible way, including the preparation margins, the finishing line, and the area beyond preparation [10,11]. Furthermore, with IOS, bleeding of the gingival margin can sometimes mask the finishing line, making it impossible for the dental technician to correctly and entirely read it. In the absence of a readable margin, the dental technician can have difficulty designing a restoration that closes and adapts perfectly to the existing prosthetic preparation. This can result in the fabrication of an incongruous prosthetic restoration [10,11]. An incongruous restoration, which presents non-optimal adaptation (for example, horizontal, vertical, or absolute marginal discrepancies), can produce a series of negative effects, such as greater accumulation or retention of plaque and therefore gingival inflammation (gingivitis) that can subsequently develop into periodontal pocket formation [17,18]; in addition, in the long term, complications such as secondary caries [19] and, in some susceptible biotypes, recessions can occur [20]. For all these reasons, the precision of a restoration does not exclusively represent the search for an optimal transition between the natural element and the prosthesis, but it is key to the longevity (or survival) of the restoration itself [2,11,17,18,19,20]. To date, numerous techniques have been proposed for the correct scanning and visualization of the margins of prosthetic preparations on natural teeth, including traditional techniques used also with conventional impressions with polyethers/polyvinylsiloxane, such as placement of one or two gingival retraction cords [21,22], the use of retraction pastes [23], gels [24] or strips [25], and the use of a laser [26]. Although with all these techniques, it is certainly possible, through proper tissue management, to obtain satisfactory optical impressions, the art of capturing the preparation margins remains a daily issue in the modern digital dental practice and a problem rather underestimated in the current literature [11]. In 2017, Mandelli et al. were the first to propose a mixed analog-digital technique to capture the subgingival margins of prepared teeth in challenging cases [11]. This technique was based on scanning with an IOS, outside of the mouth, a partial polyvinylsiloxane or polyether impression [11]. This impression was captured, the normals were inverted, and the file was substituted inside the mesh from the intraoral scanning [11]. This to allow an adequate visualization of the preparation margins, and to overcome the limits of digital impressions by using the favorable rheologic properties of conventional materials [11].

The aim of this study, that represent the development of the one published by Mandelli et al. [11], was to present a strategy for successfully capturing the margins of prepared teeth with IOS, so as to allow the dental technician to optimally visualize the margin line of preparation and thus design and fabricate clinically precise monolithic translucent zirconia crowns.

## 2. Materials and Methods

### 2.1. Patient Selection

In a period between January and December 2018, all patients who presented themselves at a private clinic to undergo fixed prosthetic rehabilitation on natural teeth were considered for possible inclusion in the present prospective clinical study. Inclusion criteria were (1) the presence of a single, partially fractured or compromised tooth, to the point of requiring prosthetic rehabilitation by means of a single crown; (2) the presence of a previous tooth-supported damaged prosthetic crown, to be replaced with a new one; (3) all adjacent teeth (mesial and distal, where present) without any prosthetic restoration; (4) age between 18 and 80 years; (5) good general health; (6) good compliance with oral hygiene; (6) willingness to adhere to the protocol of this study, which was appropriately explained in every detail, and therefore to regularly attend all scheduled work sessions. Exclusion criteria were (1) the presence of more teeth or (2) more prosthetic abutments to be rehabilitated, through a fixed prosthetic restoration (bridge or other fixed partial prosthesis); (3) the presence of a prosthetic restoration even on only one of the adjacent teeth; (4) the presence of dental implants in the reference hemiarch; (5) age <18 years or >80 years; (6) presence of systemic diseases of a chronic or inflammatory nature; (7) poor compliance with oral hygiene; (8) inability to regularly attend the appointments needed to complete the established prosthetic therapies. In accordance with the aforementioned inclusion and exclusion criteria, patients who exhibited the conditions foreseen in the inclusion criteria, and who on the contrary did not have any of the conditions listed in the exclusion criteria, were enrolled in the present prospective clinical study. These patients therefore followed the planned therapy, which consisted in all cases of rehabilitation with a single monolithic crown in translucent zirconia, obtained through a full digital CAD/CAM procedure, without the fabrication or printing of any physical model. The present study was carried out in full compliance with the principles set out in the Helsinki Declaration on experimentation on human subjects (2008 revision). Moreover, it received approval by the Ethics Committee at the Sechenov First State Medical University, Moscow, Russia.

### 2.2. Clinical and Technical Phases

**Figure 1:**
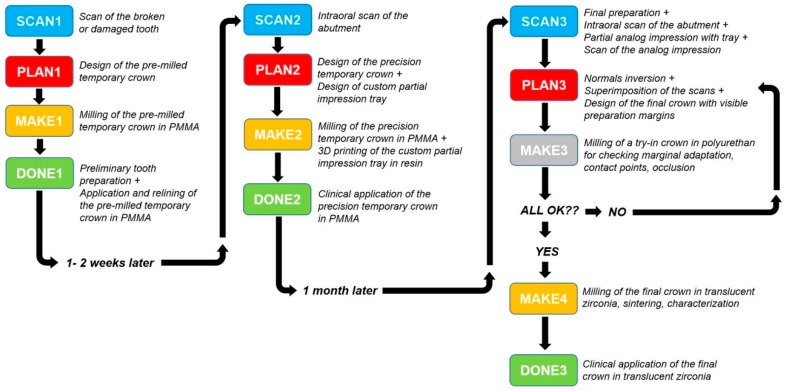
Schematic representation of all the clinical and technical phases leading to the final prosthetic restoration.

**Figure 2:**
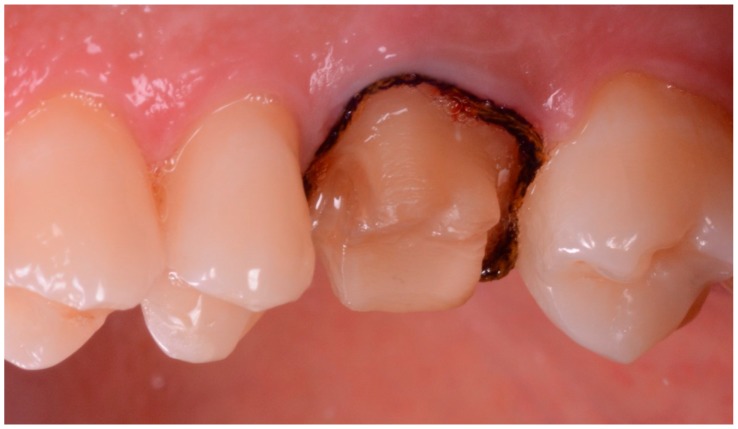
The final prosthetic preparation with the retraction cord in position.

**Figure 3:**
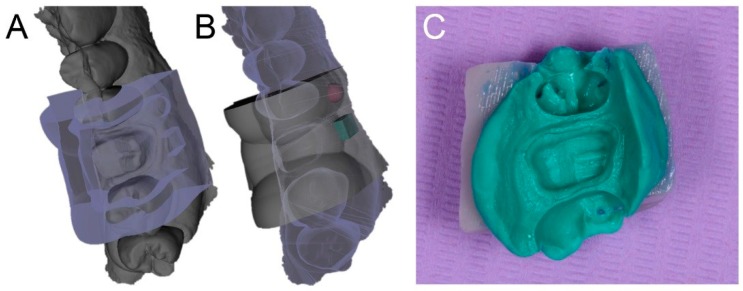
The custom partial tray designed in computer-assisted-design (CAD) software (**A**,**B**) and relined with polyvinyl siloxane light (**C**). (**A**): occlusal view of the tray with the abutment; (**B**): occlusal view of the tray and its supports; (**C**): the analog impression captured with the tray.

**Figure 4:**
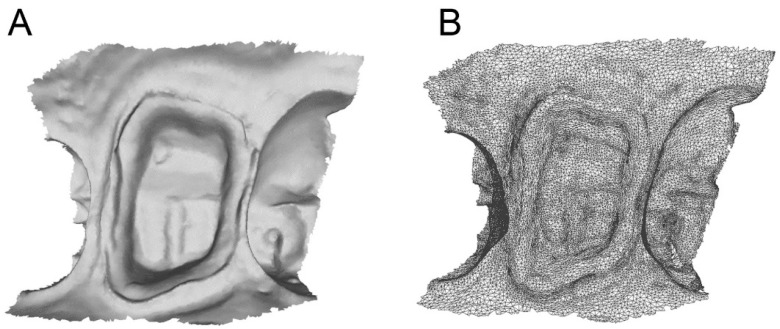
Scan of the hollow portion of the relined custom 3D printed partial tray (**A**) with mesh evaluation (**B**). (**A**): the .STL file of the preparation; (**B**): Details of the mesh with triangles.

**Figure 5:**
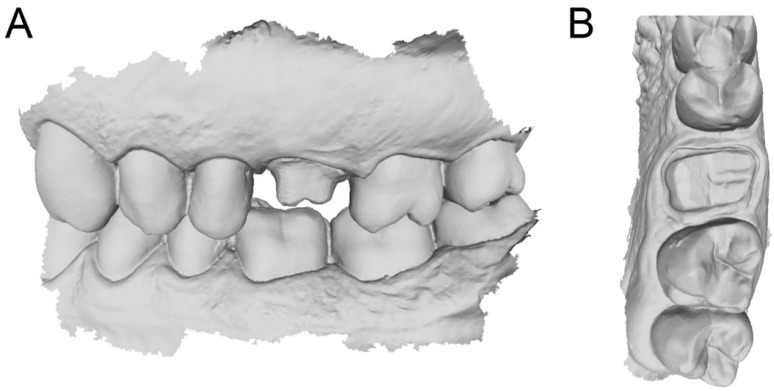
Final direct intraoral scans in occlusion (**A**) and details of the maxilla (**B**) where in some areas, the margin line is not clearly visible. (**A**): lateral view; (**B**): occlusal view.

**Figure 6:**
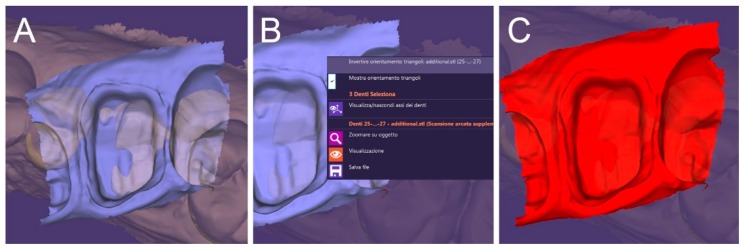
The scan of the hollow portion of the relined tray is imported in the CAD (together with the direct optical impressions captured in the patient’s mouth) (**A**) and its normals are inverted (**B**,**C**). (**A**): the additional scan is imported in the software; (**B**): inversion of the normals; (**C**): the normal have been inverted.

**Figure 7:**
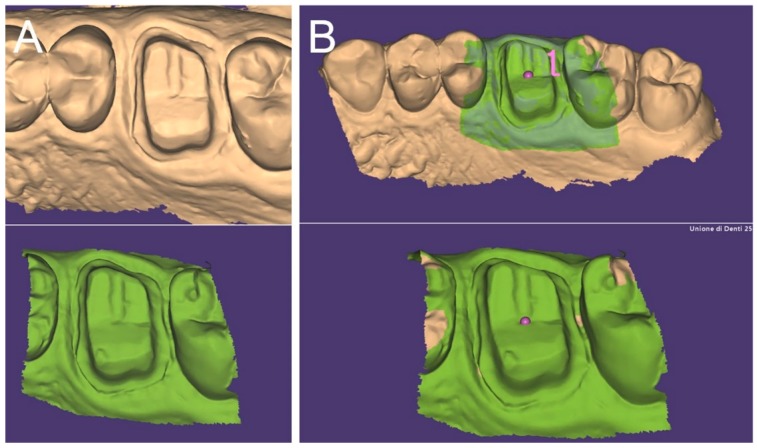
Superimposition of the two files. (**A**) The files ready for superimposition; (**B**) the superimposed files.

**Figure 8:**
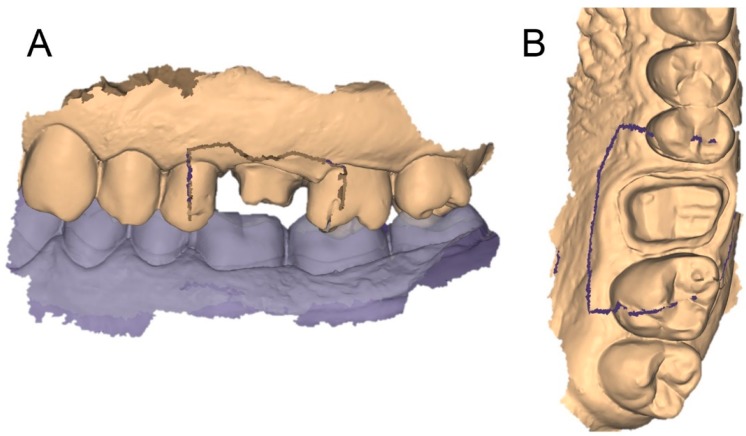
The two scans (the one of the hollow portion of the relined tray with inverted normal and the direct scan taken into the patient’s mouth) are now integrated in one single project (**A**,**B**). The margin line is now better seen, together with some details of the areas beyond; therefore, the dental technician can easily design the final crown. (**A**): lateral view; (**B**): occlusal view.

**Figure 9:**
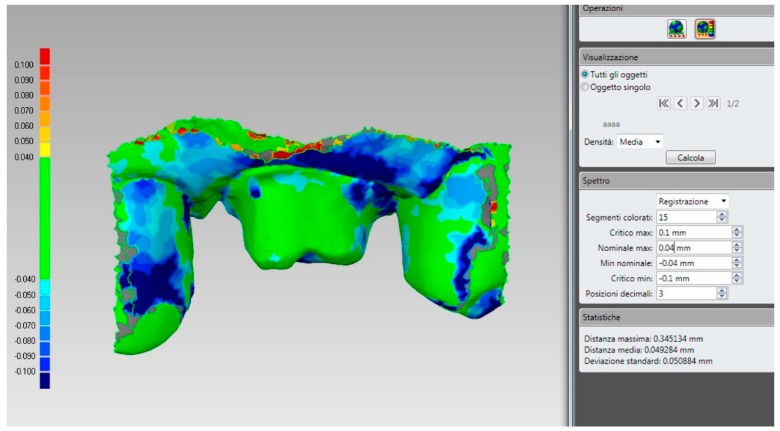
If we save the two different files (the one derived from the scan of the relined tray and the intraoral impression) in the same reciprocal position obtained after the superimposition in the CAD, and we calculate their distances in a powerful reverse-engineering software (Studio 2012, Geomagics, Morrisville, NC, USA) obtaining a colorimetric map, we can see that, as expected, the abutment has little or no deviations (green color = deviations <40 micrometers). If a deviation occurs here, it is probably related to distortion in one of the two impressions/scans. Obviously, the area of the margin line shows more deviations, because affected by the presence of tissues, since in the direct intraoral impression, the soft tissues generally cover the margins; for this reason, an overall mean deviation of 49 micrometers is registered here. However, for the aforementioned reasons, this data does not represent the quality of the superimposition, which is higher; for a fair evaluation, the margin area should be trimmed out, and only the abutments should be used for this calculation. Hence, the present superimposition technique needs further research for final validation.

**Figure 10:**
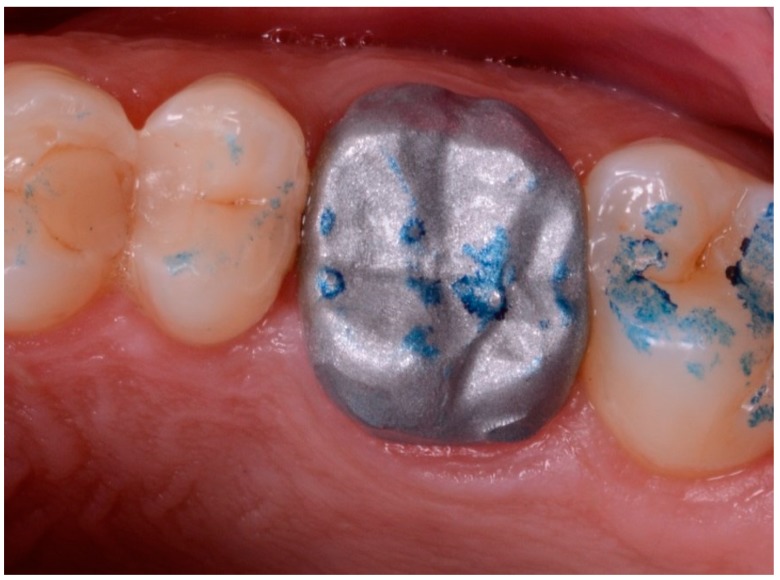
The first proof of the fit, of the marginal closure/adaptation, and of the quality of the occlusal and interproximal contacts was made using a replica of the final prosthetic restoration, milled in polyurethane. In this case, the mesial contact point was not satisfactory and had to be improved; this information was sent to the dental technician for the final CAD adaptation.

The clinical and technical phases followed a previously described path [27] and are summarized in Figure 1. During the first meeting, a preliminary scan of the hemiarch of interest was taken with an IOS (CS 3600^®^, Carestream Dental, Atlanta, GA, USA). The scan was performed in orthodontic mode, to obtain two virtual models—master model (i.e., the model with the compromised tooth to be restored with the single crown) and its antagonist. The occlusion was captured by registering the bite and the .STL files derived from this first scan were sent to CAD software (DentalCAD^®^, Exocad, Darmstad, Germany) for the design and preparation of a provisional pre-milled restoration. This provisional was milled in polymethyl-methacrylate (PMMA) using a desktop milling machine (DWX-4^®^, DGShape a Roland Company, Hamamatsu, Japan) and, after being milled and characterized, it was ready for application. The damaged tooth was then prepared preliminarily and the provisional pre-milled crown in PMMA was relined on it. After relining, the provisional was finished, taking care that the relining margins were regular; the occlusion and interproximal contact points were carefully controlled, and the crown was cemented on the prosthetic abutment with temporary cement (Tempbond^®^, Kerr, Orange, CA, USA). After a period of 1 to 2 weeks, the temporary pre-milled crown could be removed and replaced by a second, more precise PMMA provisional, to be adapted to the abutment without relining. For this purpose, the patient was referred to the clinic for the final preparation of the prosthetic abutment. The chosen preparation was horizontal (chamfer). The preparation of the abutment took place under 4.5× magnification (Zeiss^®^, Oberchoken, Germany). At the end of this procedure, a new optical impression was recorded with the same IOS (CS 3600^®^, Carestream Dental, Atlanta, GA, USA), in prosthetic mode (with higher resolution), without using any retraction method. The .STL files of the virtual models deriving from this impression (master model with prepared abutment, antagonist, and bite) were sent to CAD software (DentalCAD^®^, Exocad, Darmstad, Germany) for the design and preparation of a second, more precise temporary restoration, to be cemented directly on the prosthetic abutment without any relining. Again, this restoration was milled in PMMA with a desktop milling machine (DWX-4^®^, DGShape a Roland Company, Hamamatsu, Japan), characterized, and readied for application. Once the premilled was removed, the second provisional was applied and cemented with temporary cement (Tempbond^®^, Kerr, Orange, CA, USA) after careful control of the fit, the occlusion, and the contact points. This second provisional had to remain in the mouth for no more than 30 days before the final optical impression was taken, for the production of the final crown in monolithic translucent zirconia. The master model with the prepared abutment was, moreover, used for the design of a customized partial tray, for relining an analog impression. The procedure was as follows. The .STL file of the master model was imported into free CAD software (Meshmixer^®^, Autodesk Research, Toronto, ON, Canada). Inside this software, a customized partial tray for impression was designed. This tray was built in order to rest on the occlusal surface of the adjacent teeth (or more teeth in the same hemiarcate) for an optimal stabilization and a simple and repeatable positioning. At this level, there was a contact between the tray and the occlusal surfaces, while the whole area around the prosthetic abutment was discharged in the CAD design, so as to have a uniform and free space of 2 mm there. This design allowed the control of the relining of the impression material, obtaining an ideal support and thrust for penetration into the gingival sulcus. The customized partial impression tray was then printed by means of a desktop 3D printer (X-Fab 2000^®^, DWS Systems, Thiene, Italy) using a transparent proprietary resin (DS3000^®^, DWS Systems, Thiene, Italy) certified for intraoral use. Once removed from the printing plate, the customized tray was washed in alcohol, but it was not polymerized. Hence, after 30 days of provisionalization, the patient was recalled for the final impression. The operator checked the prosthetic preparation, working again under 4.5× magnification (Zeiss^®^, Oberchoken, Germany) and moved the prosthetic margins iuxta- or subgingivally, according to the specific clinical indications. The prosthetic preparation was always horizontal (chamfer) and took place with a retractor cord in place (Figure 2). Upon completion of the prosthetic preparation, the operator filled the customized partial 3D-printed tray with a minimum amount of polyvinyl siloxane light material. Then, immediately after removal of the retraction cord, the operator injected the polyvinyl siloxane light into the sulcus, using an injection gun, as with conventional analog impressions. He then relined the 3D-printed partial tray over the prosthetic preparation. After about 3 or 4 min, when the material was completely hardened, the tray was removed (Figure 3) and an extraoral scan of the hollow portion of it was carried out, with the same IOS (CS 3600^®^, Carestream Dental, Atlanta, GA, USA), in prosthetic mode (Figure 4). During this scan, it was important to be able to capture all details of the margin line, perfectly depicted by the light of the polyvinylsiloxane. The operator had to insist on capturing all the internal walls and the bottom of the impression. At the end of this procedure (which took place extraorally in 5 min and with the patient comfortably seated at the chair), the scan file of the impression was saved in .STL, and finally, a rapid intraoral scan of the arches was carried out. This scan took place without a retraction cord and therefore without paying excessive attention to the margin area (Figure 5). The scans of the master, antagonist, and bite models in prosthetic mode (high resolution) were then saved in .STL according to the previously described modalities. At this point, the operator had at his disposal two different optical impressions: the optical impression of the hollow portion of the relining with polyvinylsiloxane light, in a single file, extraorally captured and the patient’s models, captured intraorally. In the first impression, the details of the preparation margins were more evident because they were correctly highlighted by the impression material and able to penetrate deeply into the sulcus and read also slightly beyond. However, this impression was limited to the area of interest and therefore to the prepared abutment; it did not include other dental elements, and above all, it was reversed with respect to the second impression (i.e., it had inverted normals). In the second impression, however, the master and antagonist models were complete, with correct occlusion, but the prosthetic margins were not clearly visible. These impressions therefore had to be superimposed and fused together, in order to be able to build a unique virtual model in which the margins of the prosthetic preparations were clearly visible for the dental technician. The procedure was therefore as follows: The master and antagonist scans were imported in CAD (DentalCAD^®^, Exocad, Darmstad, Germany) into a correct occlusal relationship. Before starting the editing/cropping procedures of the models and the design of the margin, the scan of polyvinylsiloxane relining was imported too, as an “additional arch scan”, and using the appropriate tool, the normals of this scan were inverted (Figure 6). Therefore, this additional scan was superimposed on the intraoral scans of the dentate models, through the command “register scans” (Figure 7). The superimposition was made by points and surfaces, generating a colorimetric map able to give an idea of the quality of the overlap between the two files. At this point, the process was completed because the additional scan (in which the prosthetic margins were clearly visible) replaced, in the area of interest, the mesh acquired in the mouth (with the margins not visible). This fusion made it possible to eliminate the part of the scan in which the margins were not visible, replacing it with the additional scan (with clearly visible margins), integrated into a single file (Figure 8). The technician could then proceed to the CAD modeling of the final restoration, having at his disposal a single virtual model with well visible prosthetic margins. Eventually, the intraoral scan could be imported as an additional .STL file, in order to check any possible deviation or distortion between the two models (Figure 9). The proof of the fit—and of the marginal closure/adaptation—of the occlusal and interproximal contacts was made first by using a replica of the final prosthetic restoration, milled in polyurethane with a desktop milling machine (DWX-4^®^, DGShape a Roland Company, Hamamatsu, Japan). In other words, from the CAD modeling file of the final restoration, a gray polyurethane crown was obtained in CAM, which was used for the control of the marginal gaps/clinical precision, the occlusion, and the contact points, before moving to the milling of the final monolithic restoration in translucent zirconia. In short, the marginal adaptation of the polyurethane replica of the final restoration was carefully checked using magnifying glasses (Zeiss 4.5x^®^, Zeiss, Oberkochen, Germany) and physically probing the margin area with a periodontal probe (Figure 10). This procedure was performed circumferentially all around the crown, in order to intercept any possible misfit, gaps, or undercuts. The occlusion was carefully checked too, using articulating papers (Bausch Articulating Paper^®^, Bausch Inc., Nashua, NH, USA). In the case of occlusal precontact, a photograph was taken and transmitted to the dental technician, as valid information for making occlusal changes on the CAD drawing of the final modeling. Finally, contact points were checked using interdental floss. If the contacts were too weak or unsatisfactory, this information was passed to the dental technician for the appropriate modifications. After checking all these elements and making the necessary corrections, the final monolithic restoration was milled in translucent zirconia, using a 5-axis milling machine (Roland DWX-50^®^, DGShape, a Roland Company, Hamamatsu, Japan). The crown was then sintered in an oven (Tabeo^®^, Mihm-Vogt, Stutensee, Germany), characterized, and readied for cementation. Before cementing the final crown, the main outcomes of this study (i.e., the marginal adaptation of the final crown, the quality of occlusal contacts, and interproximal contact points) were verified again. In case of no issues, the final crown was cemented with a resinous cement (Bifix SE^®^, Voco GmbH, Cuxhaven, Germany), taking care to remove all possible excesses and remnants of resin before polymerization. The cementation was obtained with the light of a dental polymerization lamp. Conversely, in case of issues or misfits evidenced during this last control, the crown was not cemented and sent back to the technician.

### 2.3. Study Outcomes

There were three main outcome variables in the present study, evaluated at the delivery of the final translucent monolithic zirconia crowns. These outcome variables were: (1) the marginal adaptation of the final crown; (2) the quality of interproximal contact points; (3) the quality of occlusal contacts. All these outcome variables were carefully evaluated, in all patients, at the moment of delivery of the final crown. The marginal adaptation was evaluated by means of magnifying glasses (Zeiss 4.5x^®^, Zeiss Oberkochen, Germany) and physical probing of the margin area with a periodontal probe. The goodness of the contact points with adjacent teeth was carefully checked with dental flosses, and the absence of occlusal precontacts was controlled with the aid of articulating papers (Bausch Articulating Paper^®^, Bausch Inc., Nashua, NH, USA).

### 2.4. Statistical Analysis

All data was collected from the records of the patients consecutively enrolled in the study. Descriptive statistics were performed for the patients’ demographics (gender, age at start of the prosthetic treatment) and the location/position of the crowns. Absolute distributions were calculated for qualitative variables (marginal adaptation, quality of interproximal and occlusal contacts). Finally, means, standard deviations, medians, and 95% confidence interval (CI) were estimated for quantitative variables (patient’s age at start of the prosthetic treatment).

## 3. Results

**Figure 11:**
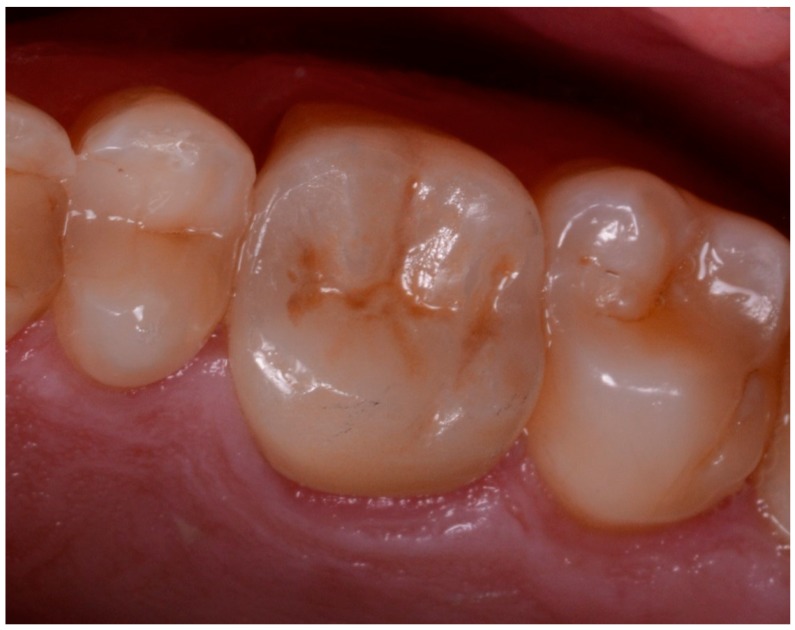
The final monolithic translucent zirconia crown after cementation.

Thirty patients (between 24 and 71 years of age, mean age 51.3 ± 11.6 years, median 51 years, CI 95% 47.2–55.4 years) who each had been restored with a single monolithic translucent zirconia crown were included in this study. Among these, only 5 were smokers. With regard to gender, there was a prevalence of male patients (18 males, 60%, vs. 12 females, 40%). However, the distribution of the prosthetic restorations was rather homogeneous, by location (16 maxillae, 53.3%, vs. 14 mandibles, 46.7%) and position (14 premolars, 46.7%, vs. 16 molars, 53.3%). At the end, the overall marginal adaptation of the monolithic crowns in translucent zirconia on the finishing line was optimal, as verified by clinical inspection and probing. In fact, only one crown presented a marked misfit (i.e., a gap on the mesial part), as verified already during the intraoral try-in with the polyurethane replica. This misfit was due to an error in the interpretation of the design of the margin line in the CAD software, due to the fact that the analog impression itself was not clearly readable in that area. This problem was reported to the dental technician, who re-designed and re-milled the try-in accordingly; at the second attempt, this issue was solved. Likewise, interproximal contact points were perfect in almost all crowns, except one that did not have the mesial contact point as verified during the try-in replica. Since a missing contact point may cause issues like food impaction in daily life, the design was sent back to the technician for modification, and no further problems were reported at this level. The most common and needed adaptations were occlusal. Precontacts were found during the try-in with polyurethane replicas, and this information was transmitted to the technician by means of clinical pictures; however, despite this and despite the modification of the CAD design, some occlusal adaptations were also necessary before the final cementation of the monolithic translucent zirconia crowns. It was, in fact, necessary to polish with zirconia brushes some points of the cusps, where there still were light pre-contacts. At the end of the study, all the monolithic crowns sat perfectly on the margins without any clinically detectable gap and were therefore cemented (Figure 11).

## 4. Discussion

In prostheses, on natural teeth, the prosthetic margins should be kept just as iuxtagingival and therefore in the sulcus, to avoid invading structures such as the periodontium (biological width) [28,29]. This is true in most cases and especially in the posterior sectors in the presence of non-discromic abutments and dental substance adequate to obtain a good retention of the restoration (particularly with single crowns) [28,29]. In most cases, there is no need to penetrate deep into the sulcus, and any bleeding during the final impression phase may be evidence of tissue suffering, resulting, for example, from an inadequate provisionalization [28,29]. However, there are some situations, for example, in the case of highly discromic abutments in the anterior areas, in which the clinician is forced to place the margins subgingivally, to avoid unpleasant chromatic effects (and an aesthetic failure of the restoration). In the same way, in the absence of adequate tissue for retention, the clinician can be forced to do the same, preparing more in depth [28,29]. The latter situation may cause issues if the instrument used is an IOS. When using IOS, the correct visualization of the margins of prosthetic preparations of natural teeth can be difficult, especially if these margins are subgingival but also where they are simply iuxtagingival (in the presence of bleeding, for example) [2,10]. In fact, as demonstrated in the literature, an IOS projects light, and this does not possess the rheological properties of conventional impression materials (polyether and polyvinylsiloxane) [2,10,11]. Conventional, analogic impression materials can be injected into the sulcus and harden inside it, “gently detaching” the soft tissues and “reading” the margin line area, accordingly [11]. This is a rather common problem for the clinician who works in a private practice and does not have the experience or skills necessary to capture an excellent optical impression using the gingival retraction systems that are currently available on the market. Despite the fact that several studies carried out by highly experienced clinicians in universities have demonstrated that the quality of marginal closures with monolithic restorations obtained with a full-digital procedure is high [12,13,14,15,16], there is a risk that the general practitioner may encounter serious difficulties in the correct use of IOS for scanning natural teeth. Consequently, the experienced clinician risks abandoning the use of IOS, claiming it does not work. The less experienced one, on the other hand, risks producing a series of restorations with a very poor marginal closure, with all the related negative consequences for the patient (sensitivity, infiltration, secondary caries of the abutments) [18,19]. Recently, great success has been achieved with a new type of prosthetic preparation, the biologically oriented preparation technique (BOPT) [30,31,32]. This preparation has spread widely, and many dentists have adopted it as the preferred way to prepare the natural abutments, with the intention of simplifying the preparation protocols. The BOPT concept represents a development of the old vertical preparation, which was used on periodontally compromised teeth with an open-flap procedure, in order to preserve dental tissue [30,31,32]. Nowadays, there are clinicians who suggest to always adopt vertical preparation with digital workflows, and specifically with IOS. They claim that eliminating the margin line eliminates the problem of reading it. In this way, processes should be greatly simplified. Unfortunately, this is a simplistic approach, which does not solve the problem at all. It is not by eliminating the preparation margins, i.e., by transforming all the preparations into vertical preparations, that an ideal closure of the definitive monolithic zirconia restoration can be obtained with a digital procedure [33]. First, the notion of being able to solve different clinical cases with a single type of preparation may be questionable because the choice of the preparation should not take place *a priori*, based on a philosophical choice, but rather on the basis of careful analysis of the clinical case with its specific indications, i.e., on a case-by-case basis; the experience of the operator is always key. Furthermore, vertical preparations do not eliminate the margin. In vertical preparations, in fact, there is an area of transition between the tissue prepared by the bur and the intact tissue; in this area, the restoration should ideally close. Finally, there are no studies in the literature that show how the type of preparation (and in particular, vertical preparation) can affect the reading ability of the IOS at iuxtagingival and subgingival levels [33,34,35]. The ability to read depends on where the margin or the closing area is placed (the deeper the margin, the worse the reading) and on the intrinsic characteristics of the scanner used, namely accuracy, acquisition resolution, tessellation, and topography [10,34,35]. In an in vitro study, Nedelcu and colleagues pointed out how the margin position has a heavy influence on the quality of the reading, even as different IOSs give significantly different readings [11]. The authors constructed an in vitro reference model with iuxta- and subgingival margin preparations, then scanned it with an industrial scanner to acquire a reference model, and finally with 7 different IOSs [10]. They then overlapped the intraoral scans with the reference scan, to evaluate the accuracy of the different scanners, with particular attention to the critical area of the margin. They finally compared all the meshes obtained, to determine in which of these the prosthetic margin (and therefore the closure line) was most easily visible (and therefore identifiable for the dental technician in CAD) [10]. Different scanners were not able to represent the margin area accurately enough, especially where the margin was subgingival [10]. Moreover, beyond the accuracy, only in the presence of an adequate number of triangles in the mesh (and, therefore, of an adequate resolution of acquisition) was it possible to visualize the margin well; however, such visualization was more difficult in subgingival areas [10]. This experiment, with all the limitations of the in vitro approach (no bleeding, no gingival crevicular fluid, no patient movements), has outlined how the reading capacity depends on the position of the margin and on the scanner used [10]. There is no scientific evidence, on the contrary, that the type of preparation (vertical vs. horizontal) can influence in some way the reading capacity of the IOS [10,32,33,34,35,36]. In the present prospective clinical study, we presented a mixed analog-digital technique for the exact capture of the margins of single tooth abutments with IOS. This technique, originally described by Mandelli et al. [11], is based on scanning the hollow portion of a partial polyvinylsiloxane or polyether impression. The normals are inverted, and the file is replaced inside the mesh from the intraoral scanning. This is basically the recovery of the partial impression technique described long ago by the Italian Casartelli (technique of the copper ring) [37], integrated into a digital workflow. In the present study, following a similar clinical protocol, the dental technician could model final crowns that presented a perfect adaptation on the margin line, in almost all 30 patients treated. Similar results were found for the interproximal contact points, while, on delivery, for zirconia crowns, some (minimal) occlusal adjustments were necessary. Such adjustments, which are often needed with IOS, are likely to depend on the still-to-be-perfected mechanism of bite or chewing capture. However, despite the good clinical results obtained in this group of patients, there are caveats to be issued regarding this approach, as well as some elements still to be clarified and limitations to be highlighted. First, the CAD technique presented here is complex and requires highly skilled clinicians and technicians with deep knowledge in digital dentistry to care about; it involves a certain number of steps that cost time and effort. Moreover, from qualitative control of the coherence between the file of the abutment derived from the direct intraoral scan and that generated by the inversion of the normals of the analog impression, some differences or deviations emerge, especially in the most coronal area. Although these deviations can be verified and normalized in CAD, it is evident that the capture of the deepest part of the internal, hollow portion of the analog impression can be difficult for the IOS. Then, the results reported in the present study probably depend on the IOS used, which in other applications has shown high accuracy [7], despite having a limited acquisition resolution. Therefore, they cannot be generalized to other scanners, which need to be tested in this specific application. Finally, and more than anything else, this technique is not fully digital, and the present clinical study has limitations because it describes the resolution of only 30 cases. Further studies with a larger number of patients and restorations are necessary in order to be able to draw more specific conclusions on the reliability of the present technique with the present IOS. In addition, this technique should be tested and verified in the case of multiple natural abutments, i.e., for the restoration of bridges or partial prostheses that may be more difficult [38] and may present more issues. Clearly, a system capable to directly scanning through the soft tissues would be recommended. However, while waiting for the development of an IOS able to read through soft tissues, it might be useful for clinicians who have a scanner with a low acquisition resolution to become familiar with this technique.

## 5. Conclusions

In fixed prosthodontics, particularly when working with natural teeth, the marginal fit and therefore the clinical precision of restorations is key. An incongruous restoration, which possesses non-optimal adaptation (with misfits or discrepancies) can determine a series of negative effects, such as plaque retention, gingival inflammation, periodontal pocket formation or recessions, as well as secondary caries. For this reason, even in the full digital workflow and working with monolithic zirconia crowns, it is essential to design and fabricate restorations that perfectly fit and close on the margin line of the prosthetic abutments. Although the clinical precision of a monolithic crown not only depends on the scan but is the result of a series of subsequent steps (design, milling, sintering), the first thing to do with IOS is to obtain an accurate optical impression, on which the dental technician can reliably identify the margin line, in order to correctly design the closures in the CAD. Unfortunately, the capture of the margins of prosthetic preparations with IOS can be complex, especially in the case of bleeding, or when they are sub-gingival; in fact, light does not possess the rheological properties of conventional impression materials, which gently “displace” soft tissues, harden inside the sulcus, and record every detail of the area of interest. In the present clinical study, we have presented an experimental mixed analog-digital protocol for the optimization of the capture of the margin line of the prosthetic preparation with an IOS. This protocol is based on the scanning with IOS, outside the mouth, of a partial custom 3D-printed tray, physically relined on the abutment with polyvinylsiloxane light. The impression of the hollow portion of this tray is captured, the normals are inverted, and this file is superimposed on the mesh obtained directly in the mouth from an intraoral scan, replacing it. This is in order to allow the dental technician to better visualize the prosthetic preparation margins. In our present clinical study, based on 30 patients rehabilitated with a single monolithic translucent zirconia crown, the present analog-digital protocol for capturing the margins of prepared single abutments was compatible with the fabrication of clinically precise restorations. However, several aspects related to this mixed protocol still need to be elucidated, and further studies on a larger sample of patients and on different types of prosthetic restorations (such as bridges or fixed partial prostheses) are certainly needed to draw more specific conclusions about the reliability of this technique.

## Data Availability

The datasets are available from the author upon reasonable request.

## Abbreviations

IOS	Intraoral scanner
CAD	Computer Aided Design
CAM	Computer aided Manufacturing
PMMA	Polymethylmethacrylate
